# Metabolic Dysregulation and Female Infertility: A Systematic Review of Hormonal and Reproductive Outcomes From Recent Clinical Trials

**DOI:** 10.7759/cureus.90887

**Published:** 2025-08-24

**Authors:** Nimrah Ashraf, Asma Qayyum, Rabia Bashir, Sara Zubair Ahmed, Misbah Shafiq, Munaza Batool, Muhammad Sahil, Mariam N Kakar

**Affiliations:** 1 Obstetrics and Gynaecology, Gweru Provincial Hospital, Gweru, ZWE; 2 Internal Medicine, Dow International Medical College, Karachi, PAK; 3 Obstetrics and Gynaecology, Bukayriyah General Hospital, Al Bukayriyah, SAU; 4 Obstetrics and Gynaecology, Burjeel Specialty Hospital, Sharjah, ARE; 5 Internal Medicine, Baqai Medical University, Karachi, PAK; 6 Internal Medicine, Shahida Islam Medical Complex, Lodhran, PAK; 7 Internal Medicine, Rawalpindi Medical University, Rawalpindi, PAK; 8 Internal Medicine, Mayo Hospital, Lahore, PAK; 9 Obstetrics and Gynaecology, Bolan University of Medical & Health Sciences, Quetta, PAK

**Keywords:** hormonal imbalance, infertility, insulin resistance, intervention strategies, metabolic syndrome, pcos, reproductive outcomes, women's health

## Abstract

This systematic review explores the influence of metabolic and immunological interventions on hormonal and fertility outcomes in infertile women with metabolic disorders such as polycystic ovary syndrome (PCOS), insulin resistance, obesity, and metabolic syndrome. The review synthesizes findings from rigorously selected randomized and crossover trials published between 2015 and 2024, focusing on interventional approaches aimed at improving reproductive and endocrine parameters. Studies were screened and assessed using the PRISMA (Preferred Reporting Items for Systematic Reviews and Meta-Analyses) guidelines, and relevant outcomes included ovulation, oocyte quality, pregnancy rates, and changes in hormonal markers, such as insulin, luteinizing hormone (LH), follicle-stimulating hormone (FSH), and estradiol. The analysis reveals that metabolic dysregulation significantly alters reproductive outcomes and that targeted interventions, ranging from pharmacological agents and dietary modifications to experimentally induced metabolic states, can have measurable effects on hormonal profiles and fertility. While some interventions demonstrated clear improvements in ovulatory and metabolic markers, others primarily highlighted associations rather than causality. Despite variations in sample sizes and methodological designs, the review underscores the need for integrated therapeutic strategies addressing both metabolic and hormonal dysfunction in this population. The findings support a multifaceted approach to managing infertility in women with metabolic disturbances, emphasizing the potential for tailored interventions to optimize both endocrine and reproductive health.

## Introduction and background

Female infertility is a growing global health concern, affecting approximately 8-12% of reproductive-aged women worldwide. While the causes of infertility are multifactorial, ranging from anatomical and infectious to hormonal and environmental, metabolic disorders, operationally defined in this review as conditions that disrupt systemic metabolic homeostasis and hormonal regulation (including polycystic ovary syndrome (PCOS), insulin resistance, obesity, metabolic syndrome, and dyslipidemia), have emerged as critical but underrecognized contributors to reproductive dysfunction [1]. These conditions can significantly disrupt the hypothalamic-pituitary-ovarian (HPO) axis, leading to impaired ovulation, abnormal sex hormone profiles, and decreased fertility potential [2,3].

PCOS, one of the most prevalent endocrine disorders among women of reproductive age, is a prototypical example of metabolic and reproductive interplay [4]. Characterized by hyperandrogenism, ovulatory dysfunction, and polycystic ovarian morphology, PCOS is also closely associated with insulin resistance, obesity, and dyslipidemia. These metabolic disturbances exacerbate hormonal imbalances, contributing to menstrual irregularities and subfertility. Similarly, metabolic syndrome, defined by a cluster of conditions including central obesity, hyperglycemia, hypertension, and dyslipidemia, has been shown to impair endometrial receptivity and oocyte quality, adversely affecting outcomes of both naturally conceived pregnancies and assisted reproductive technologies (ART) such as in vitro fertilization (IVF) [5,6].

Emerging clinical trials have begun to explore the mechanistic links between metabolic dysfunction and reproductive outcomes, as well as the therapeutic potential of interventions targeting metabolic health. These include dietary modifications, insulin-sensitizing agents like metformin and GLP-1 receptor agonists, antioxidant supplementation, and lifestyle interventions [7]. Additionally, studies are increasingly investigating the direct endocrine consequences of metabolic stress, including altered luteinizing hormone (LH) pulsatility, pituitary responsiveness, and levels of leptin, ghrelin, and sex steroids [8].

Despite this growing body of research, the data remain fragmented across specialties and disease models. A comprehensive synthesis of recent clinical trials is essential to better understand how metabolic dysregulation contributes to infertility and how these insights can be translated into effective, fertility-enhancing interventions. To ensure clinical relevance and capture advances in therapeutic strategies, this review was limited to studies published in the past 10 years (2015-2024), reflecting the most up-to-date diagnostic criteria, intervention modalities, and fertility outcomes. The objective of this systematic review is to critically evaluate and synthesize findings from recent clinical trials that assess the impact of metabolic disorders on hormonal regulation and fertility outcomes in women, with a focus on interventional and mechanistic studies. By consolidating this evidence, the review aims to clarify the pathophysiological links and highlight therapeutic opportunities to improve reproductive outcomes in metabolically affected populations.

## Review

Materials and methods

Study Design and Reporting Framework

This study was conducted as a systematic review in accordance with the PRISMA (Preferred Reporting Items for Systematic Reviews and Meta-Analyses) 2020 guidelines [9]. The design adhered to a structured approach using the PICO (Population, Intervention, Comparator, Outcome) framework [10], which enabled a focused and comprehensive review of clinical evidence. The objective was to examine the effects of metabolic and autoimmune disorders on hormonal profiles and reproductive outcomes in women experiencing infertility.

Eligibility Criteria

Studies were eligible for inclusion if they investigated infertile women with metabolic conditions such as PCOS, insulin resistance, obesity, or metabolic syndrome and evaluated the effects of metabolic or immunological interventions on hormonal or fertility outcomes. Acceptable study designs included randomized controlled trials (RCTs), crossover trials, and clinical intervention studies published between 2015 and 2024. Eligible studies were required to assess at least one hormonal parameter or reproductive outcome, such as ovulation, oocyte quality, fertilization rate, or clinical pregnancy. Studies were excluded if they were conducted on animals, focused on non-reproductive populations, were not interventional in design, or failed to report relevant hormonal or fertility-related outcomes. Only English-language studies were included to ensure the accuracy and reproducibility of findings.

Search Strategy

A comprehensive literature search was conducted using three major databases: PubMed, Scopus, and Web of Science. The search strategy incorporated a combination of medical subject headings (MeSH) and free-text terms, including but not limited to "polycystic ovary syndrome", "insulin resistance", "female infertility", "metabolic syndrome", "reproductive hormones", "ovulation", "in vitro fertilization", "metformin", "liraglutide", and "clinical trials". Boolean operators such as AND and OR were employed to optimize the sensitivity and specificity of the search results. Filters were applied to limit the search to human studies published in English. Reference lists of the included articles were also hand-searched to identify additional studies that met the inclusion criteria.

Study Selection

Two reviewers independently screened the titles and abstracts of the retrieved articles to assess relevance and eligibility based on the predefined criteria. Full-text versions of potentially eligible studies were then reviewed in detail. Any disagreements during the selection process were resolved through consensus after discussion, ensuring a systematic and unbiased approach to study inclusion.

Data Extraction

Data were extracted from the included studies using a structured data collection form developed specifically for this review. The extracted information included the study design, sample size, participant characteristics, intervention type and duration, comparator details, and primary outcomes assessed. Particular attention was given to hormonal parameters such as LH, follicle-stimulating hormone (FSH), estradiol, insulin, testosterone, and anti-Müllerian hormone (AMH), as well as reproductive outcomes such as ovulatory status, oocyte retrieval, fertilization rates, and pregnancy rates. Data extraction was conducted independently by two reviewers to maintain accuracy and reproducibility.

Risk of Bias Assessment

To assess the methodological quality of the included RCTs, the Cochrane Risk of Bias tool (RoB 2.0) [11] was employed. This tool evaluates five key domains: the randomization process, deviations from intended interventions, missing outcome data, measurement of outcomes, and selection of reported results. Each domain was judged as presenting low risk, some concerns, or high risk of bias. Overall risk of bias was also categorized based on the collective assessment of these domains. This evaluation enabled a critical appraisal of the internal validity of the evidence included in this review.

Data Synthesis and Analysis

Given the heterogeneity in intervention modalities, participant characteristics, and reported outcomes, a quantitative meta-analysis was deemed inappropriate. Therefore, a qualitative synthesis was conducted. The included studies were thematically grouped based on the nature of the metabolic intervention or autoimmune factor involved, as well as the specific outcomes measured. To address heterogeneity beyond the decision not to conduct a meta-analysis, we employed a structured narrative comparison, organizing studies by intervention type (e.g., pharmacological, dietary, experimental metabolic models) and target population (e.g., PCOS, obesity, metabolic syndrome). This approach allowed us to highlight convergent findings within similar categories while also noting where results diverged across different populations or methodologies. Given the limited number of eligible trials, formal subgroup or sensitivity analyses were not feasible; however, potential sources of heterogeneity were explicitly acknowledged during interpretation, ensuring that our conclusions reflect both the strengths and the variability of the available evidence.

Results

Characteristics of the Selected Studies

**Figure 1:**
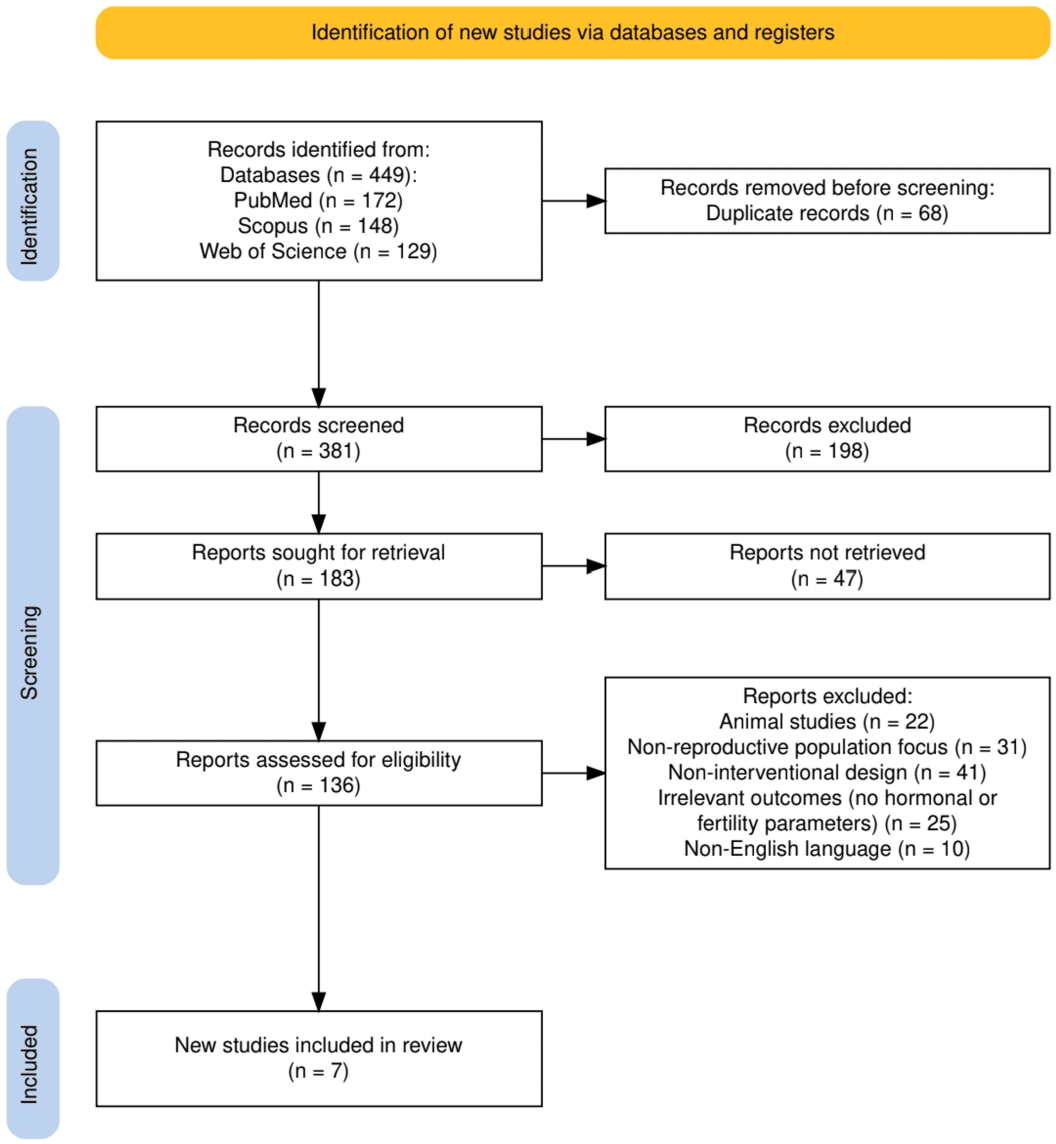
The PRISMA flowchart represents the study selection process. PRISMA: Preferred Reporting Items for Systematic reviews and Meta-Analyses

A total of 449 records were identified through three electronic databases: PubMed (n = 172), Scopus (n = 148), and Web of Science (n = 129). After removing 68 duplicate records, 381 studies underwent initial screening, of which 198 were excluded based on title and abstract. Of the remaining 183 full-text articles sought for retrieval, 47 could not be obtained, leaving 136 for eligibility assessment. Following a detailed review, 129 studies were excluded for reasons including the use of non-human subjects, non-reproductive populations, non-interventional designs, the absence of relevant outcomes, or the use of a non-English language. Ultimately, seven studies met the inclusion criteria and were incorporated into this systematic review. The detailed flow of the selection process is illustrated in Figure 1.

Study Selection Process

Table 1 provides a comprehensive summary of the seven studies included in this systematic review, detailing the study designs, target populations, metabolic conditions investigated, interventions, comparators, and both hormonal and fertility-related outcomes. While the studies varied in methodology and scope, all explored the interplay between metabolic dysfunctions, such as PCOS, obesity, insulin resistance, or induced metabolic states, and reproductive or hormonal health in infertile women. Interventions ranged from pharmaceutical agents, such as metformin and liraglutide, to dietary modifications and metabolic infusions, with outcome measures encompassing key hormonal markers and reproductive endpoints, including ovulation, oocyte quality, pregnancy rates, and live birth rates. These studies collectively offer valuable insights into how metabolic and immunological interventions can influence fertility outcomes, as summarized in Table 1.

**Table 1:** Summary of clinical trials evaluating metabolic and immunological interventions on hormonal and fertility outcomes in infertile women. PCOS: Polycystic Ovary Syndrome; MetS: Metabolic Syndrome; HOMA-IR: Homeostatic Model Assessment for Insulin Resistance; QUICKI: Quantitative Insulin Sensitivity Check Index; LH: Luteinizing Hormone; FSH: Follicle-Stimulating Hormone; GnRH: Gonadotropin-Releasing Hormone; AUC: Area Under the Curve; IVF: In Vitro Fertilization; s.c.: Subcutaneous; OHSS: Ovarian Hyperstimulation Syndrome; SHBG: Sex Hormone-Binding Globulin

Study (Author, Year)	Study Design	Population (n, Age)	Metabolic Disorder	Intervention	Comparator	Hormonal Outcomes	Fertility Outcomes
Jabarpour et al., 2024 [12]	Triple-blind RCT	n = 58, infertile women with PCOS	PCOS	Astaxanthin 12 mg/day for 8 weeks	Placebo	Fasting insulin, HOMA-IR, QUICKI	Not directly assessed
Salamun et al., 2018 [13]	Randomized open-label trial	n = 28, infertile obese PCOS women, mean age 31.1 ± 4.75 yrs	PCOS + Obesity	Metformin 1000 mg BID + Liraglutide 1.2 mg/day s.c. for 12 weeks	Metformin 1000 mg BID	Not reported	IVF pregnancy rate per embryo transfer, cumulative pregnancy rate over 12 months
He et al., 2019 [14]	Secondary analysis of multicenter RCT	n = 1508 infertile women with PCOS; 410 with MetS	PCOS ± Metabolic Syndrome	Not an intervention trial (observational within RCT)	Comparison: MetS vs non-MetS within PCOS cohort	Peak estradiol, insulin resistance markers (HOMA-IR)	Duration of infertility, gonadotropin use, oocyte yield, embryo number, cumulative live birth rate, OHSS rate
Chang et al., 2019 [15]	Secondary analysis of RCT (PCOSAct)	n = 936 women with PCOS and baseline homocysteine data	Hyperhomocysteinaemia (HHCY), Metabolic Syndrome	Not an interventional arm	Comparison: HHCY group vs. normal HCY; MetS group vs. non-MetS	LH, FSH, LH/FSH ratio, estradiol, free testosterone, SHBG, fasting glucose	Ovulation rate, conception rate, pregnancy rate, pregnancy loss rate, live birth rate
Santoro et al., 2021 [16]	Randomized crossover trial	n = 15 eumenorrheic, lean women; median age 32 yrs; BMI ~21.9 kg/m²	Induced metabolic syndrome (via insulin + lipid infusion)	6-hour insulin + lipid infusion in early follicular phase	Saline infusion	LH pulse amplitude, mean FSH, GnRH-stimulated LH/FSH AUC	Not directly assessed
Becker et al., 2015 [17]	Randomized controlled trial	n = 26 overweight/obese infertile women; IVF candidates	Obesity, insulin resistance	Hypocaloric low glycemic index/load diet for 12 weeks	Control diet	Leptin, ghrelin, insulin, HOMA-IR, reproductive hormones	Number of oocytes retrieved, pregnancy rate (IVF + spontaneous), live births
McDonald et al., 2022 [18]	Randomized crossover trial	n = 12 healthy, normal-weight, eumenorrheic women	Induced hyperlipidemia and hyperinsulinemia (reprometabolic model)	Euglycemic insulin + lipid infusion (Intralipid + heparin)	Saline + heparin	FSH, LH (↓), TSH, PRL, GH, fT4, T3, cortisol, IGF-1, leptin (↑), adiponectin, creatinine	Not directly assessed

Quality Assessment

The risk of bias assessment, as summarized in Table 2, evaluates the methodological rigor of each included study across five critical domains based on the Cochrane Risk of Bias 2.0 tool. Most studies demonstrated a low risk of bias in the randomization process, outcome measurement, and deviations from intended interventions. However, some concerns were identified in studies involving open-label designs, secondary analyses, or small sample sizes, particularly in the domains of selective reporting and missing outcome data. Overall, four studies were deemed to have "some concerns," largely due to issues in reporting or exploratory subgroup analyses, while the remaining three studies were rated as having a low risk of bias across all domains. To assess whether these concerns might have altered our synthesis, we considered the impact of excluding the four "some concerns" studies. Since the main trends, such as improvements in insulin sensitivity with pharmacological or dietary interventions and the adverse influence of metabolic syndrome on fertility outcomes, were consistently observed in the low-risk studies as well, the overall interpretations did not materially change. While we did not conduct a formal sensitivity analysis due to the small number of included trials, the robustness of findings across both low- and some-concern studies suggests that our conclusions remain reliable. This assessment strengthens the credibility of our synthesis, ensuring that the conclusions drawn are based on methodologically sound evidence.

**Table 2:** Risk of bias assessment of the included clinical trials using the Cochrane RoB 2.0 tool. RoB 2.0: Revised Cochrane Risk of Bias Tool for Randomized Trials; RCT: Randomized Controlled Trial; PCOS: Polycystic Ovary Syndrome; MetS: Metabolic Syndrome

Study (Author, Year)	Study Design	Domain 1: Randomization Process	Domain 2: Deviations From Intended Interventions	Domain 3: Missing Outcome Data	Domain 4: Measurement of Outcome	Domain 5: Selection of Reported Result	Overall Risk of Bias
Jabarpour et al., 2024 [12]	Triple-blind RCT	Low risk	Low risk	Low risk	Low risk	Low risk	Low risk
Salamun et al., 2018 [13]	Randomized open-label trial	Some concerns	Some concerns (open-label may affect behavior)	Low risk	Low risk	Some concerns	Some concerns
He et al., 2019 [14]	Secondary analysis of RCT	Low risk	Low risk (no new intervention)	Low risk	Low risk	Some concerns (exploratory outcomes)	Some concerns
Chang et al., 2019 [15]	Secondary analysis of RCT	Low risk	Low risk	Some concerns (subgroup attrition not clear)	Low risk	Some concerns	Some concerns
Santoro et al., 2021 [16]	Randomized crossover trial	Low risk	Low risk	Low risk	Low risk	Low risk	Low risk
Becker et al., 2015 [17]	Randomized controlled trial	Low risk	Low risk	Some concerns (small sample size)	Low risk	Some concerns	Some concerns
McDonald et al., 2022 [18]	Randomized crossover trial	Low risk	Low risk	Low risk	Low risk	Low risk	Low risk

Discussion

The intersection of insulin resistance and reproductive dysfunction is a defining hallmark of PCOS, especially in the context of coexisting obesity. Multiple studies in this review demonstrate that interventions targeting insulin resistance, such as astaxanthin supplementation [12], liraglutide combined with metformin [13], and low glycemic index diets [17], correlate with measurable improvements in insulin metrics (e.g., reductions in fasting insulin and homeostatic model assessment for insulin resistance (HOMA-IR)) and improved reproductive outcomes. For instance, liraglutide and metformin co-therapy yielded a higher cumulative pregnancy rate over 12 months compared to metformin alone, highlighting the metabolic-reproductive axis. This aligns with mechanistic pathways wherein IR disrupts HPO signaling, leading to hyperandrogenism, disrupted folliculogenesis, and ovulatory dysfunction. These findings underscore the importance of addressing metabolic dysregulation as a core strategy for improving fertility in PCOS patients, especially considering the high global prevalence of obesity among reproductive-aged women.

Beyond PCOS, the broader construct of metabolic syndrome, which includes central adiposity, dyslipidemia, and insulin resistance, directly influences assisted and natural conception. Chang et al. [15] and He et al. [14] provide compelling statistical evidence that MetS is associated with decreased oocyte yield, lower conception rates, and increased pregnancy loss. In Chang et al.'s cohort [15], women with hyperhomocysteinemia and metabolic syndrome had lower live birth rates and higher miscarriage rates, pointing toward endothelial dysfunction and oxidative stress as contributing mechanisms. Furthermore, the study by He et al. [14] found that women with metabolic syndrome required higher gonadotropin doses and had reduced embryo numbers, underscoring how metabolic dysfunction impairs ovarian responsiveness even under controlled stimulation. These results highlight that metabolic syndrome is not merely a comorbidity; it is a pathophysiological driver of reproductive inefficiency. Tailoring fertility treatments to mitigate these metabolic burdens could enhance reproductive outcomes in both natural and assisted settings.

Emerging evidence increasingly positions metabolic dysfunction as a central contributor to reproductive impairment in women. This systematic review builds upon previous observational research, such as the Nurses' Health Study II and data from the Rotterdam PCOS cohort, which highlight associations between obesity, insulin resistance, and subfertility. Notably, while existing meta-analyses have confirmed lifestyle interventions can improve ovulatory patterns, very few have synthesized interventional trials assessing hormonal biomarkers and fertility outcomes in tandem [19,20]. The present review fills this gap by compiling robust randomized trials and crossover studies that reveal both biochemical and clinical fertility improvements following targeted metabolic interventions. For instance, studies by Santoro et al. [16] and McDonald et al. [18] provide mechanistic depth showing that even short-term metabolic disruption can acutely suppress gonadotropin secretion, an effect not previously highlighted in traditional endocrinology texts.

The clinical implications of these findings are profound. Intervening at the level of metabolic dysfunction, through pharmacological agents such as liraglutide or through dietary modification, has demonstrable effects not only on insulin sensitivity but also on oocyte yield and pregnancy success rates [21]. Given that conditions like PCOS and obesity often coexist with metabolic syndrome, early correction of these disturbances may enhance the efficacy of ART [22]. As a result, clinicians should consider integrating metabolic screening, including HOMA-IR and lipid profiles, into routine infertility workups. With regard to the timing of IVF or ovulation induction, our suggestion to optimize metabolic parameters first should be interpreted cautiously. The included trials primarily report short- to medium-term improvements in insulin resistance, hormonal balance, and ovulatory outcomes, but few provide longitudinal evidence linking pre-IVF metabolic correction to live birth rates. Therefore, this recommendation is based on short-term outcomes and a mechanistic rationale rather than definitive long-term trial data, underscoring the need for prospective studies to confirm its validity [23].

This review offers several strengths, including its exclusive focus on recent interventional trials and its incorporation of hormonal endpoints alongside fertility metrics. The inclusion of mechanistic crossover trials provides unique physiological insights that are often missing from meta-analyses focused solely on conception rates. However, limitations remain. Many of the included studies had small sample sizes, which limits statistical power and generalizability. Heterogeneity across interventions, ranging from astaxanthin to insulin infusions, posed challenges for quantitative synthesis. Moreover, most trials did not assess long-term outcomes such as cumulative live birth rates, and data on autoimmune contributors to infertility remain sparse, despite initial intentions to include such studies.

Despite promising findings, key research gaps persist. Larger, multicenter RCTs are urgently needed to evaluate how metabolic interventions impact long-term fertility and live birth outcomes, particularly in diverse populations. There is also a glaring void in studies evaluating autoimmune mechanisms, such as thyroid autoimmunity or systemic lupus erythematosus, as modulators of hormonal balance in infertile women. Additionally, research should extend beyond the classic phenotype of obese PCOS to include lean women with subtle metabolic abnormalities or unexplained infertility [24,25]. Exploring combinations of immunomodulatory and insulin-sensitizing therapies may open new therapeutic frontiers in reproductive medicine.

## Conclusions

This systematic review underscores the pivotal role of metabolic health in regulating female reproductive function, offering compelling evidence that hyperinsulinemia and hyperlipidemia can disrupt key hormonal pathways, even with short-term exposures. By synthesizing interventional trials and mechanistic studies, our findings illuminate how metabolic derangements selectively impair the hypothalamic-pituitary-gonadal axis while sparing others, highlighting a cell-type-specific vulnerability. Importantly, these mechanistic disruptions translated into measurable clinical effects across the included trials: reductions in insulin resistance (e.g., decreased HOMA-IR and fasting insulin) were consistently associated with improved ovulation and conception rates, while adverse metabolic profiles such as metabolic syndrome and hyperhomocysteinemia correlated with lower oocyte yield, increased miscarriage risk, and reduced live birth outcomes. Thus, the biological plausibility established through endocrine pathways is directly reflected in the quantitative fertility outcomes observed.

The review not only bridges a critical gap in the literature but also provides a strong rationale for integrating metabolic screening and management into infertility workups and treatment protocols. Ultimately, our study advocates for a paradigm shift: reproductive endocrinology must evolve beyond ovarian stimulation to embrace metabolic restoration as a foundational element of fertility care. This insight holds immense significance, especially in an era marked by rising obesity and insulin resistance, and lays the groundwork for more effective, personalized reproductive interventions.
